# Combination of scanning probe technology with photonic nanojets

**DOI:** 10.1038/s41598-017-03726-5

**Published:** 2017-06-14

**Authors:** Martí Duocastella, Francesco Tantussi, Ali Haddadpour, Remo Proietti Zaccaria, Andrea Jacassi, Georgios Veronis, Alberto Diaspro, Francesco De Angelis

**Affiliations:** 1 Nanophysics, Istituto Italiano di Tecnologia, Via Morego 30, 16063 Genoa, Italy; 2 School of Electrical Engineering and Computer Science, Louisiana State University, Baton Rouge, LA 70803 USA; 3 Center for Computation and Technology, Louisiana State University, Baton Rouge, Louisiana 70803 USA

## Abstract

Light focusing through a microbead leads to the formation of a photonic nanojet functional for enhancing the spatial resolution of traditional optical systems. Despite numerous works that prove this phenomenon, a method to appropriately translate the nanojet on top of a region of interest is still missing. Here, by using advanced 3D fabrication techniques we integrated a microbead on an AFM cantilever thus realizing a system to efficiently position nanojets. This fabrication approach is robust and can be exploited in a myriad of applications, ranging from microscopy to Raman spectroscopy. We demonstrate the potential of portable nanojets by imaging different sub-wavelength structures. Thanks to the achieved portability, we were able to perform a detailed optical characterization of the resolution enhancement induced by the microbead, which sheds light into the many contradictory resolution claims present in literature. Our conclusions are strongly supported by rigorous data analysis and by numerical simulations, all in perfect agreement with experimental results.

## Introduction

In the quest for a fundamental understanding and control of the building blocks of nature, several optical approaches have been developed capable of resolving or manipulating deep sub-wavelength structures. A widely used strategy consists in the activation/deactivation of fluorophores or photo-initiators to confine chemical processes down to a size as small as 40 nm, as in super-resolution microscopy^1, 2^ or two-beam direct laser write lithography^3, 4^. Unfortunately, these methods are intrinsically linked to the photophysics of the relatively scarce materials that can be optically deactivated. Alternatively, near-field effects can focus light beyond the limits dictated by diffraction thanks to the collection of evanescent waves. Due to the fast decay of these waves, a probe must be typically placed in close proximity to the targeted sample, such as in near-field scanning optical microscopy (NSOM)^5^. Despite reported resolutions of 12 nm, the distance between probe and sample is extremely critical and difficult to control, and it generally suffers from poor signal to noise ratio (SNR). This has prompted the development of methods capable of efficiently projecting evanescent waves into the far field, ranging from superlenses^6^, solid immersion lenses (SIL)^7, 8^ or microbeads^9^. This latter is particularly interesting due to the wide availability of microbeads with different size or refractive index, and the ease of implementation of this approach.

Focusing light through a microbead with a radius of 2–50 µm results in the formation of an elongated jet with a sub-wavelength diameter, referred to as photonic nanojet^10^. Extensive literature on this subject has been recently published that demonstrate the potential of these nanojets for photopolymerization^11^, surface nanopatterning^12^ or enhanced resolution imaging in bright-field^9, 13^, wide-field^14^ and even confocal microscopy^15^. However, the use of nanojets suffers a serious drawback inherent to its simplicity. Usually, microbeads are placed on the surface of interest with no control. As a consequence, not a single microbead may lay on top of the object to be imaged/modified, rendering the concept unusable. An apparent simple solution to this problem is to increase the number of microbeads on the sample. At high concentrations, though, microbeads tend to aggregate and can axially overlap, deteriorating optical performance. Even if laser irradiation of self-assembled microbeads prepared on top of a surface can generate periodic nanopatterns^16^, the minimum distance between the fabricated nanostructures is still limited by the microbead diameter. Therefore, controlling the position of nanojets on user-defined areas is key for the further development of this technology.

Previous attempts to control the position of nanojets include the preparation of arrays of microbeads embedded in an elastomeric matrix^13, 17^, the translation of the bead with optical tweezers^12, 18^, a micro-needle attached to a hydraulic micromanipulator^19^, propulsion by local catalytic reactions^20^, or the use of a glass micropipette placed on an XYZ stage^21^. However, the displacement of the elastomer with respect to the surface, the complexity of the setup required for optical trapping, or the fragility of glass and potential micropipette breakage render these approaches difficult to implement in practice. Recently, a microbead glued to a cantilever has been used to scan the nanojet on top of extended structures^22, 23^, but potential contamination of the microbead by glue and consequent degradation in imaging performance can limit this implementation. Arguably, because of the lack of control in the positioning of microbeads, a complete characterization of the optical performance of nanojets for high-resolution imaging is still missing. Indeed, such analysis requires the use of standardized resolution criteria appropriate for partially coherent systems^24^. Given the small field of view obtained with microbeads, this demands, in turn, for the ability to translate microbeads. Furthermore, different parameters have been used to define spatial resolution in these systems, including the distance between the edge of two features or the distance between maximum and minimum contrast. Thus, there exists a large disparity in the data regarding attainable resolution using microbeads for imaging applications, with claimed values ranging from 25 nm to more than 300 nm in the visible range. Even if the full width at half maximum (FWHM) of the point spread function (PSF) has been recently measured by de-convolution methods^13, 17, 23, 25, 26^, some a priori knowledge was required (shape of the PSF). In fact, this has been the subject of recent controversy^27^. As a result, a full description of the optical response of a microbead has not yet been provided.

In this article, we present an alternative method to easily translate a photonic nanojet to targeted positions on a sample based on mounting a microbead on a tipless atomic force microscope (AFM) cantilever using electrostatic forces. A XYZ piezoelectric stage enables the fine adjustment of the nanojet position, whereas measurement of the deflection of the AFM cantilever can be used to *in-situ* quantify the distance between microbead and sample. By integrating the microbead-cantilever system into a microscope, the portable nanojet can be exploited to enhance the focusing capabilities of traditional microscope objectives. The simplicity of our approach makes it an easily accessible and powerful technique in relevant fields ranging from plasmonics^28^ to micro or nano Raman spectroscopy^29^. Among the different applications, we demonstrate the potential of portable nanojets by imaging different extended structures. Because of this portability, we were able to perform for the first time a detailed optical characterization of the spatial frequency response of a microbead coupled to a microscope by means of the modulation transfer function (MTF), and to determine the corresponding frequency cutoff (strict definition of spatial resolution) for different focusing objectives. Finite difference time domain (FDTD) simulations are in good agreement with experiments.

## Results

**Figure 1:**
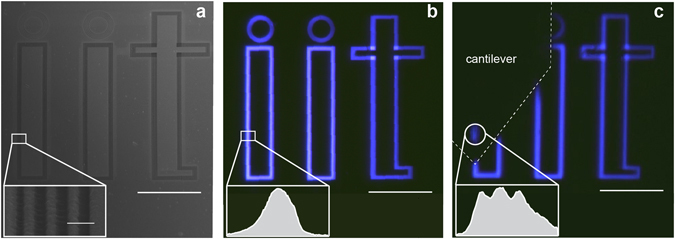
Traditional imaging versus microbead imaging. (**a**) SEM micrograph of a structure consisting of the “iit” logo. As shown in the inset, each letter consists of equally spaced lines with a periodicity of 380 nm. Scale bar is 20 µm, inset scale bar is 500 nm. (**b**) Optical image of the structure using 450 nm transmitted illumination with a 50×, 0.5 NA. Scale bar is 20 µm. The periodic lines of each letter cannot be resolved, as shown in the intensity plot of the inset. (**c**) Virtual image obtained using the microbead. Scale bar is 20 µm. In this case, the image formed through the microbead can resolve the periodic lines (inset). Note that due to the limited field of view of the microbead, only 3 lines are visible.

Initially, we evaluated the capacity of our approach to translate and position a microbead across an extended sample. In this case, we used a structure consisting of the letters “iit” fabricated on a gold-coated glass substrate by FIB lithography. Notably, each letter contained a sub-structure comprised of lines with a periodicity of 380 nm (Fig. 1a). An image acquired using a 50x long working distance objective (NA 0.5, working distance 10.3 mm, Olympus LMPLANFL) with transmitted 405 nm light from a blue LED is presented in Fig. 1b. We selected this particular color since it offered higher contrast and spatial resolution than white light. As expected, at these conditions the “iit” letters can be clearly resolved, but not so the lines within each letter. Note that the diffraction limit in this case is ~405 nm, above the periodicity of the sub-structure. When using the microbead, though, the periodic lines can be resolved (Fig. 1c). In this case, the distance between microbead and sample was maintained at 50 nm. This separation was found to be the optimal tradeoff between image contrast and possibility to freely translate the nanojet while preventing the adhesion of the microbead to the sample (see Supplementary Figure 1 and Supplementary Movie 1). The image was collected with the objective focal plane located 4.5 µm below the microbead base, thus the formed image was virtual. More importantly, as shown in Supplementary Movie 2, the AFM cantilever enables to easily translate the microbead across the “iit” letters, providing a locally magnified image with enhanced resolution at user-defined positions.

**Figure 2:**
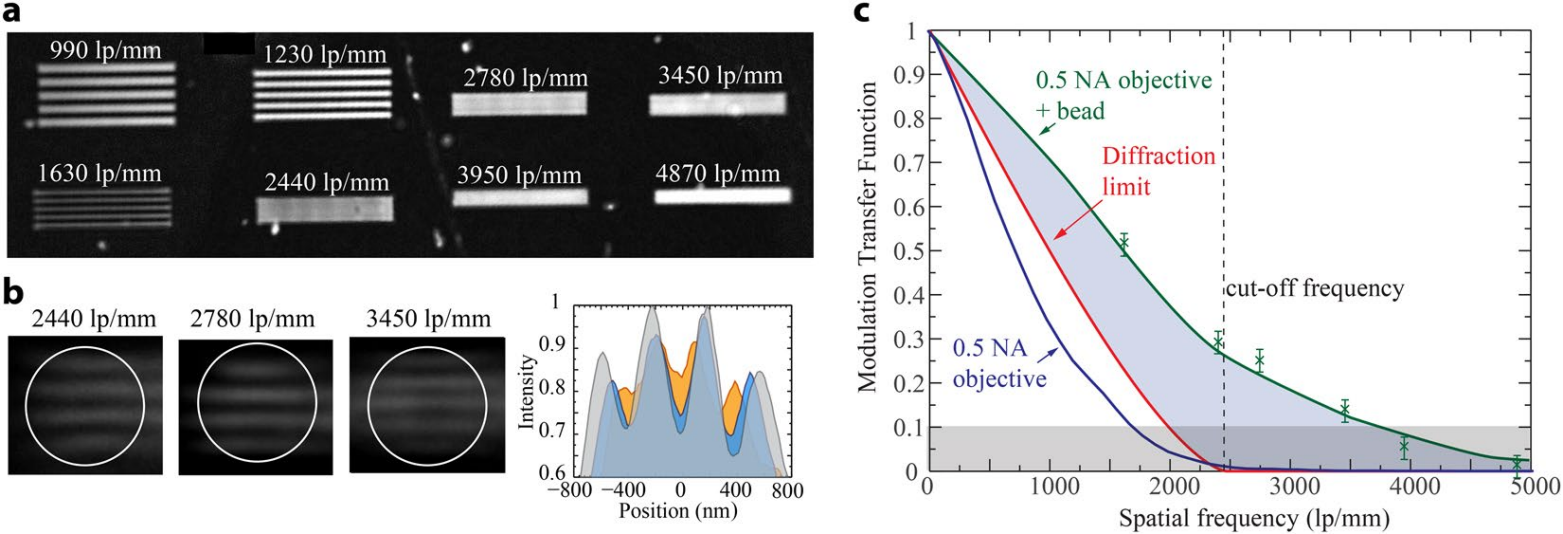
Optical response in a microbead coupled to a bright-field microscope. (**a**) Optical micrograph acquired with the 50× objective of gratings imaged without the microbead. (**b**) Details of three gratings imaged through the microbead. Note that these frequencies could not be resolved without the microbead. The corresponding intensity profiles (normalized with respect to 2440 lp/mm) are also shown. (**c**) Frequency response of the microscope with and without microbead. The response from a diffraction limited system has also been included. The gray area indicates the 10% MTF criterion used to define the maximum attainable resolution. The highlighted purple area indicates the enhancement in resolution achieved with the microbead with respect to a perfect diffraction-limited system with a 0.5 NA. The cut-off frequency indicates the Abbe resolution limit for a 0.5 NA system.

The possibility to locate the microbead at targeted locations enables to perform a detailed optical characterization of the resolution enhancement induced by the nanojet. To this end, we first characterized the optical frequency response of our microscope without a microbead. Note that an optical microscope can be rigorously defined as a band-pass filter, where diffraction dictates the particular frequency response. In this experiment, we imaged a customized calibration target fabricated with FIB lithography and consisting of gold gratings on glass with periodicities ranging from 100 to 1000 nm (Supplementary Figure 2). This calibration target can be considered as the high resolution equivalent of the 1951 USAF test chart typically used to measure resolution in partially coherent systems. Other test charts, such as a Siemens start, could be used for the same purpose^24^. Given the small field of view of the microbead, though, a target composed of periodic straight lines was considered more adequate. An image of the calibration target acquired in transmission mode with a wavelength of 405 nm is presented in Fig. 2a. Only structures with a grating period below 1630 line-pairs (lp) per mm can be distinguished. To further refine this measurement, we captured an image of a slant sharp edge^30^. From this image we could directly extract the edge response of our microscope, and by differentiation calculate the line spread function (LSF). Interestingly, the LSF is the 1D equivalent of the PSF of a microscope (image of a point source or impulse response), and thus from its Fourier transform we could retrieve the frequency response of our system, also known as MTF (Fig. 2c). The MTF intuitively indicates contrast and it illustrates the behavior of a microscope as a filter, with the cutoff frequency (maximum resolution) conventionally considered at a 10% of the MTF (the Rayleigh resolution criterion corresponds to a 9% of the MTF). Our particular microscope objective presented a maximum resolution of 580 nm, which corresponds to an effective NA of 0.42. For comparison, the optical performance of an ideal diffraction-limited system with NA = 0.5 was plotted in Fig. 2c. The corresponding cutoff frequency in this case was 2*NA*/*λ* ~ 2470 lp/mm. As expected, the ideal microscope presented a significant better performance at high frequencies than our microscope. This can be attributed to imperfections in the design of “real” microscope objectives as well as to the inherent noise in any optical system.

We repeated the above optical characterization in the case of using a microbead for imaging. To avoid any potential interference caused by the cantilever during image formation, experiments were performed by detaching the microbead from the cantilever at targeted locations on the different gratings. The presence of the cantilever, though, did not seem to affect image formation, neither did increasing the distance between microbead and structure to 50 nm. Indeed, the structures resolved in the case of using the cantilever, see Supplementary Movie 3, were the same as without it (Fig. 2b). Remarkably, each microbead formed a virtual image at a distance of 4.5 µm below the sample surface, with a magnification factor of 2.8. Even if higher magnifications could be obtained by moving the objective focal plane further below the surface (up to a distance of about 9 µm), at these positions the contrast was poorer while no higher spatial frequencies could be resolved, indicating a simple effect of “empty magnification”, namely magnification without gain in resolution. Importantly, the microbead enables to resolve frequencies not accessible with the regular microscope, as shown in Fig. 2b. Indeed, a grating with a spacing up to 3450 lp/mm could be clearly resolved. The corresponding MTF calculated using the slanted edge method is plotted in Fig. 2c. Notably, the range of accessible frequencies with the microbead was significantly extended compared to the regular or even diffraction-limited microscope objective used, with a maximum resolution of about 260 nm. Considering the Rayleigh criterion, this value indicates a 2.3 factor enhancement in the effective numerical aperture of our system, from 0.42 NA to an equivalent 0.95 NA objective. A similar behavior was observed with 2 additional objectives (20 × 0.4 NA and 100 × 0.8 NA). In both cases, the microbead produced an improvement in spatial resolution, but with different resolution enhancement factors. The results are summarized in Table 1. Particular attention should be given to two different scenarios. First, when the initial NA of our microscope objective is already high (>0.9), the effects of the microbead become less apparent. In fact, the maximum resolution attainable with the microbead was limited to about 260 nm, even for the highest NA objectives tested. Second, given a low NA objective, it may not be possible to fully exploit the intrinsic resolution enhancement of the microbead. In other words, a low NA objective may cutoff the high frequencies that the microbead inherently can transfer into the far field. This helps explaining the lower effective NA of the 20× objective when using the microbead. Indeed, in this case the initial objective NA was 0.3 (cutoff frequency of 1200 lp/mm), which implies that for an image magnified by a factor of 2.8, the corresponding cutoff frequency would be 2.8 × 1200 = 3400 lp/mm, which is equivalent to a 0.81 NA. This value is in good agreement with the effective NA obtained with the microbead (Table 1). We can interpret the observed trends in resolution enhancement by considering the microbead to act as a lens with a fixed numerical aperture of around 0.95. Thus, the simple introduction of the microbead can effectively turn a regular microscope into a high NA system. Moreover, to optimize resolution enhancement, the microscope objective should have a NA low enough to allow the microbead to significantly increase the optical performance of the microscope, but sufficiently high to be able to resolve the highest frequencies that the microbead can couple into the far field.

**Table 1:** Characterization of the effective NA of the different objectives used (10% MTF criterion), and the effective NA when imaging through the microbead.

	Effective NA	Microbead effective NA	Enhancement
20 × 0.4 NA	0.30	0.80	2.7
50 × 0.5 NA	0.42	0.95	2.3
100 × 0.8 NA	0.69	0.97	1.4

The resolution enhancement is also included.

**Figure 3:**
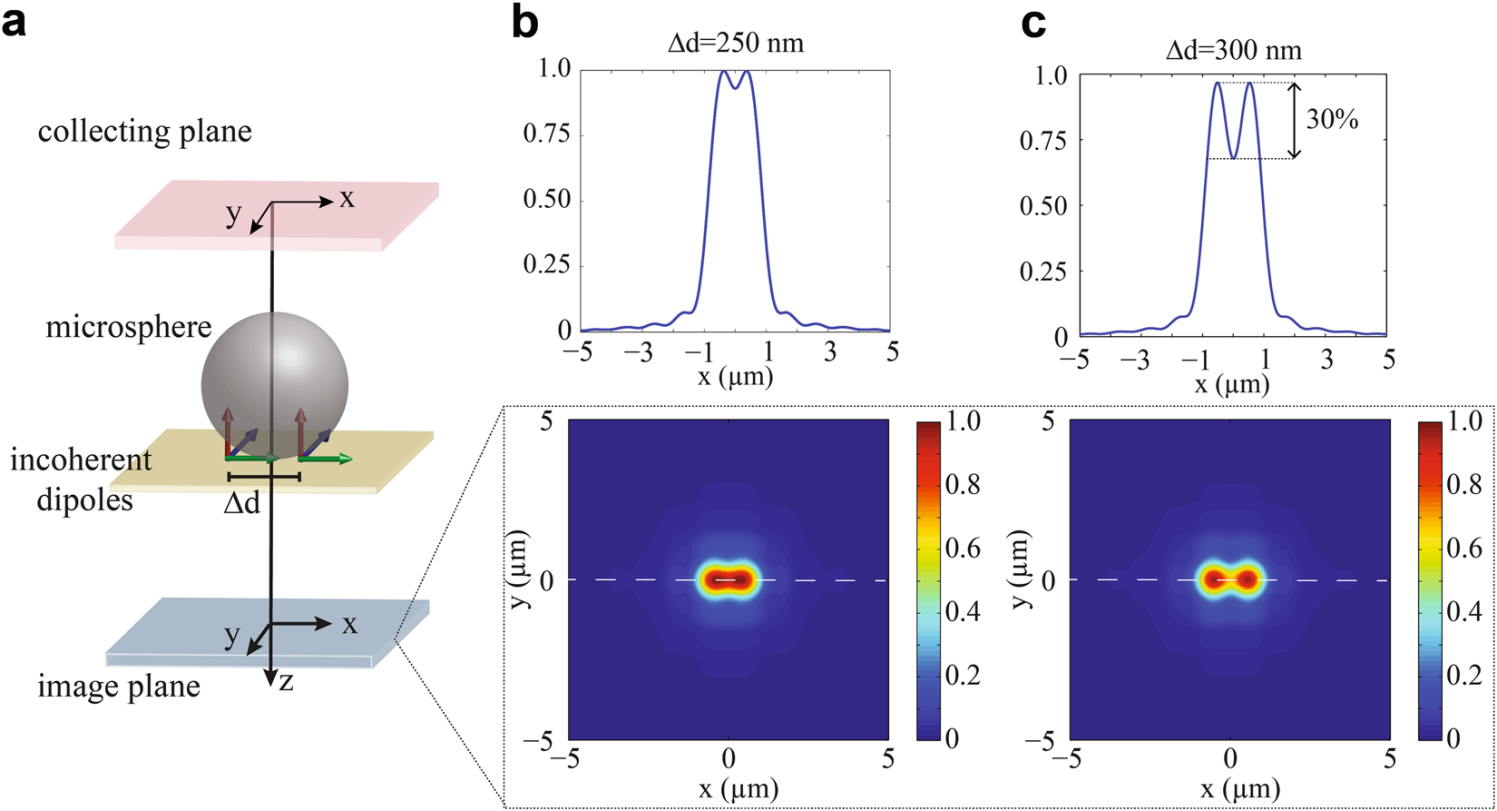
FDTD simulations of a model system for microbead image formation. (**a**) Scheme of the simulated layout, with two incoherent dipoles placed in contact with the microbead and separated a distance Δd. The electromagnetic field generated by the dipoles is calculated in the collecting plane and back-propagated. The position of maximum intensity is considered the image plane. (**b**) Normalized intensity profile and corresponding intensity colormap for two dipoles separated 250 nm and (**c**) 300 nm. Only the latter can be resolved.

To validate the idea of the microbead as a high NA lens, we performed three dimensional FDTD simulations (Lumerical Solutions) of a model system consisting of two incoherent dipoles oscillating in XYZ with a wavelength of 405 nm and placed in close proximity to a silica microbead, as similarly done in previous works^31^. The diameter of the microbead (refractive index 1.46) was set to be 4.6 µm while air was chosen as background material. Perfect Matched Layer (PML) absorbing boundary condition was used for the entire simulation window. Furthermore, convergence analysis was performed in order to guarantee solutions with an error below 5%. In more detail, the electromagnetic field generated by the dipoles was calculated at the collecting plane (5.35 µm above the dipoles), namely after passing through the microbead. Afterwards, the so calculated field was back-propagated upon removing the microbead, as illustrated in Fig. 3a, in order to simulate the formation of a virtual image. The position of maximum intensity of this field was considered to be the virtual image plane^32^. As it can be observed in Fig. 3b, dipoles separated by 250 nm could not be resolved using the Rayleigh criterion. Note that the location of the image plane was at about 4.5 µm from the dipoles plane with a magnification factor equal to 3.1, in close agreement with experiment. By increasing the separation distance of the dipoles above 250 nm (Fig. 3c), they become resolvable, as experimental results demonstrated. Therefore, the anticipated behavior of the microbead as an optical element with an effective NA of about 0.95 is confirmed by the FDTD simulations.

## Discussion

The values of frequency cutoff reported here are in contrast with super-resolution claims from previous works. Indeed, our results indicate that with a 4.6 µm silica microbead and 405 nm illumination, the maximum attainable resolution with the microbead is 260 nm, which does not break the diffraction limit of high NA immersion objectives (using the Rayleigh criterion, 1.2*λ*/2.8 ~ 175 *nm*). Importantly, in most existing literature a strict definition of spatial resolution is not used. For instance, a common parameter used to quantify resolution is the 100 nm width of the stripes of Blu-ray disks^9, 21, 25, 33–36^. Because these stripes are periodically separated from each other a typical distance of 200 nm, the parameter that defines resolution in this case is related to the total periodicity of the structure (~300 nm), as convolution with different size PSF shows^37^. This stems directly from the definition of resolution as the minimum distance at which two structures can be recognized. In fact, an isolated point or line emitter with sub-diffraction size can be distinguished in any optical system provided enough contrast (definition of PSF or LSF), but this does not imply that the system resolution is given by the emitter size^38^. It is worth noting that other structures with deep sub-wavelength periodicities have been resolved using microbeads, including 50 nm^15^, 100 nm^9^ and ~160 nm^17, 25^. We tried to image structures of similar size with our system but could not resolve them. We believe that several effects can account for this. First, the use of microbeads in combination with other imaging modalities, such as laser scanning confocal microscopy (LSCM), could result in further resolution improvement^9^. Indeed, provided a large enough photon budget, LSCM is already a super-resolution approach when the pinhole is below 1 Airy Unit^39^. Second, microbeads of a higher refractive index material (n = 1.9), or with a larger size (50 µm) and partially immersed in a fluid are expected to have a higher effective NA^40^. Finally, the effects of the particular substrate used^41^ (i.e. refractive index, plasmonic coupling, etc.) or the excitation of the electromagnetic modes^42^ could also affect the ability of microbeads to resolve a given structure. Hence, strong attention must be paid when claiming to reach a particular resolution using microbeads for imaging applications.

In conclusion, the coupling of an AFM cantilever with a microbead is an efficient and simple method to easily position nanojets on top of targeted areas on a substrate. The portable nanojet strategy presented here can be easily implemented in multiple applications where enhanced optical focusing can be advantageous, including imaging, Raman spectroscopy or photopolymerization. Thanks to the achieved nanojet portability, we provided a detailed characterization of the optical enhancement produced by microbeads for high-resolution imaging. As our results demonstrate, microbeads enable to extend the frequency response of traditional optical systems, but at conditions herein, not beyond the limits achievable with high NA systems based on immersion (NA > 1). Within this context, a microbead is a simple and affordable alternative to traditionally expensive immersion objectives or other super-resolution modalities. Furthermore, the integration of microbeads with an AFM system opens the door to correlative approaches for combining optical characterization tools with different AFM modalities (e.g. force mapping, topography), which could help getting further insights about the intrinsic properties of materials.

## Methods

**Figure 4:**
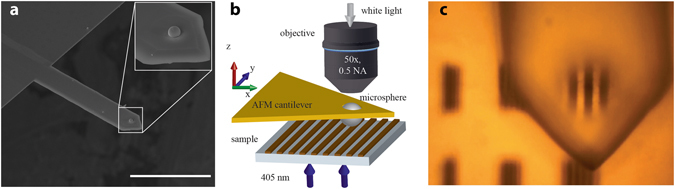
System for translating the microbeads and characterizing the optical response of portable nanojets. (**a**) SEM micrograph of a tip-less AFM cantilever with a microbead electrostatically (Van der Waals interaction) attached to it. Scale bar is 100 µm. (**b**) Scheme of the optical setup used for enhanced imaging with the microbead. The system could be operated in either reflection or transmission modes. In any case, partially coherent light was used (white light or 405 nm wavelength) and the virtual image formed was collected with a microscope objective. (**c**) Virtual image of a grating formed in reflection mode using a microbead.

A scheme of the microbead positioning system is presented in Fig. 4. An AFM system provided with a XYZ piezoelectric actuator (WiTec Alpha 300 RA) was integrated into a bright-field microscope that could be operated in reflection as well as in transmission mode. In order to position microbreads on targeted positions of a sample, we used a specially designed AFM cantilever in which 3.5 µm holes were drilled by means of focused ion beam (FIB) lithography (Helios FEI Dual Beam SEM-FIB). After placing the cantilever into the AFM holder of our microscope, we proceeded with the trapping and positioning of individual beads. To this end, we selected a single microbead from a reservoir prepared by drop casting. We then placed the hole of the cantilever on top of the bead to be captured, and moved the cantilever axially until getting in contact with the microbead. Electrostatic forces held the bead within the hole and allowed the displacement of the bead/cantilever system to the area of interest. Importantly, we could also detach the microbead on a given point of interest by firmly pressing the bead against the substrate. This is a key difference with respect to previous works using glue to attach the microbeads^22, 23^. The displacement of the cantilever was monitored in real time by means of the microscope camera (ImagingSource DFK72BUCO2). In all experiments, we used silica microbeads with a diameter of 4.7 µm (Banglabs CS019), refractive index of 1.46), as commonly used in literature^9, 34, 35^.

## Electronic supplementary material

Supplementary Information

Supplementary Movie 1

Supplementary Movie 2

Supplementary Movie 3
