# Supplementary material for: Combination of scanning probe technology with photonic nanojets
Source: Sci Rep. 2017 Jun 14;7:3474. doi: 10.1038/s41598-017-03726-5 (PMC5471276; doi:10.1038/s41598-017-03726-5)
Supplement: Supplementary file 1 — Supplementary Information [file 41598_2017_3726_MOESM1_ESM.pdf]

Supplementary Information: Combination of scanning probe technology with photonic nanojets

Martí Duocastella[1,*], Francesco Tantussi[1,*], Ali Haddadpour[2,3], Remo Proietti Zaccaria[1], Andrea Jacassi[1], Georgios Veronis[2,3], Alberto Diaspro[1], Francesco De Angelis[1]

1- Nanophysics, Istituto Italiano di Tecnologia, Via Morego 30, 16063 Genoa, Italy
2- School of Electrical Engineering and Computer Science, Center for Computation and Technology (CCT), Louisiana State University, Baton Rouge, LA 70803, USA
3- Center for Computation and Technology, Louisiana State University, Baton Rouge, Louisiana 70803, USA.

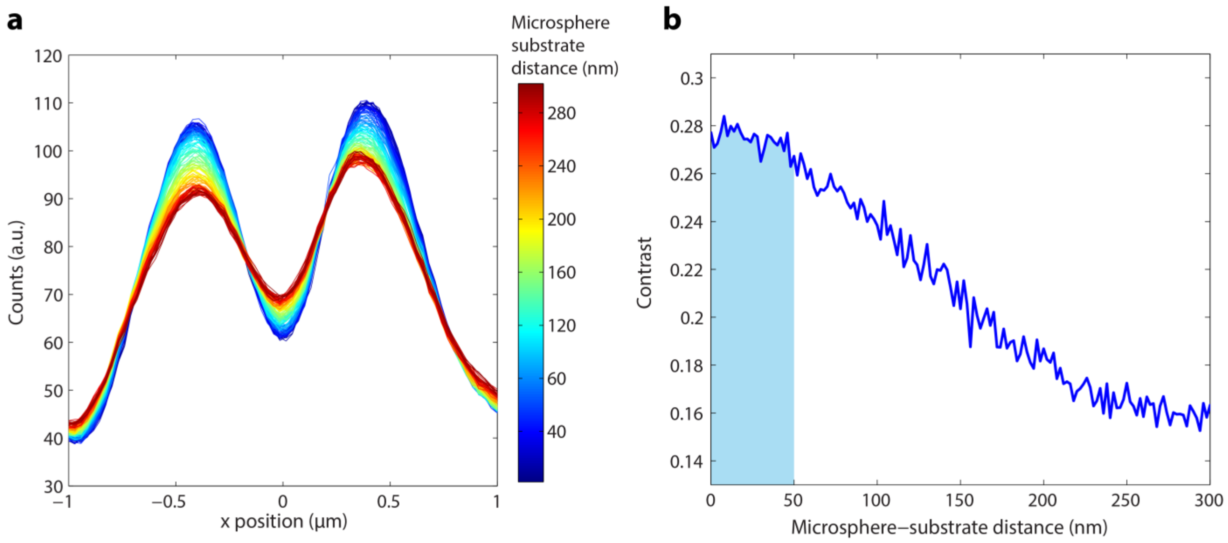

Figure S1. Characterization of the imaging performance of the microsphere as it is separated from the substrate. a) Line intensity profile of images displayed in Supplementary Video 1. A structure consisting of a gold grating with a periodicity of 800 nm was imaged at different microsphere-substrate distances (using the AFM feedback control). For each image, the corresponding intensity profile was plotted and displayed with a different color. Note that a distance of 0 nm indicates contact of the microsphere with the substrate . b) Plot of the contrast function versus microsphere-substrate distance obtained from Figure S1a. The contrast function is defined as $(I_{max} - I_{min})/(I_{max} + I_{min})$, where I_{max} is the average intensity of the 2 bright peaks and $I_{min}$is the minimum intensity between peaks. Up to a distance of around 50 nm, no significant degradation in contrast is observed. Moreover, at this distance the microsphere could be freely moved while avoiding contact with the substrate. Thus, 50 nm represented the optimal distance between microsphere and substrate.

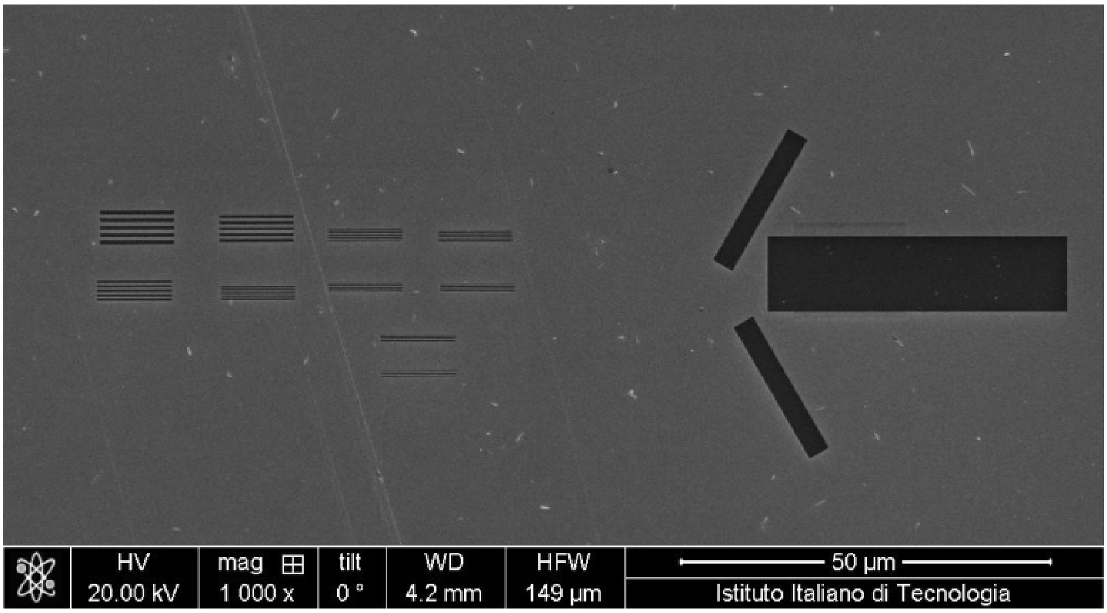

Figure S2. SEM image of the gratings used for the optical characterization of SMM.

Supplementary Movie 1. Experiment to determine the optimal separation between microsphere and cantilever. The microsphere, attached to the cantilever, was translated at 2 nm steps toward the substrate from an initial distance of 950 nm until reaching contact.

Supplementary Movie 2. Figure of the calibration target used to obtain the microbead optical response.

Supplementary Movie 3. Videos of the portable nanojet scanned over different structures.
